# Cytotoxic Effects of Pinnatane A Extracted from *Walsura pinnata* (Meliaceae) on Human Liver Cancer Cells

**DOI:** 10.3390/molecules23112733

**Published:** 2018-10-23

**Authors:** Nurhisyam Zakaria, Mohamad Azrul Mahdzir, Mahfuzah Yusoff, Norhafiza Mohd Arshad, Khalijah Awang, Noor Hasima Nagoor

**Affiliations:** 1 Institute of Biological Sciences, Faculty of Science, University of Malaya, Kuala Lumpur 50603, Malaysia; nurhisyam2309@gmail.com; 2 Department of Chemistry, Faculty of Science, University of Malaya, Kuala Lumpur 50603, Malaysia; mazrulm89@gmail.com (M.A.M.); khalijah@um.edu.my (K.A.); 3 Centre for Foundation Studies in Science, University of Malaya, Kuala Lumpur 50603, Malaysia; mahfuzahyusoff@um.edu.my; 4 Centre for Research in Biotechnology for Agriculture (CEBAR), University of Malaya, Kuala Lumpur 50603, Malaysia; hafizaarshad@um.edu.my; 5 Centre of Natural Products and Drug Discovery (CENAR), University of Malaya, Kuala Lumpur 50603, Malaysia

**Keywords:** anti-cancer, apoptosis, cell cycle arrest, necrosis, triterpene

## Abstract

Background: Pinnatane A from the bark of *Walsura pinnata* was investigated for its anti-cancer properties by analyzing the cytotoxic activities and cell cycle arrest mechanism induced in two different liver cancer cell lines. Methods: A 3-(4,5-Dimethyl-2-thiazolyl)-2,5-diphenyl-2H-tetrazolium bromide (MTT) assay was used to analyze the pinnatane A selectivity in inducing cell death in cancer and normal cells. Various biological assays were carried out to analyze the anti-cancer properties of pinnatane A, such as a live/dead assay for cell death microscopic visualization, cell cycle analysis using propidium iodide (PI) to identify the cell cycle arrest phase, annexin V-fluorescein isothiocyanate (annexin V-FITC)/PI flow cytometry assay to measure percentage of cell populations at different stages of apoptosis and necrosis, and DNA fragmentation assay to verify the late stage of apoptosis. Results: The MTT assay identified pinnatane A prominent dose- and time-dependent cytotoxicity effects in Hep3B and HepG2 cells, with minimal effect on normal cells. The live/dead assay showed significant cell death, while cell cycle analysis showed arrest at the G_0_/G_1_ phase in both cell lines. Annexin V-FITC/PI flow cytometry and DNA fragmentation assays identified apoptotic cell death in Hep3B and necrotic cell death in HepG2 cell lines. Conclusions: Pinnatane A has the potential for further development as a chemotherapeutic agent prominently against human liver cells.

## 1. Introduction

Treatment of hepatocellular carcinoma with chemotherapeutic drugs tested in randomized controlled trials has shown only moderate survival benefit for intermediate grade tumour and poor first-line treatment for advanced diseases [1,2,3]. Currently, sorafenib, a multi-target kinase inhibitor, is the recommended Food and Drug Administration (FDA)-approved drug to improve survival by controlling tumour progression in patients with advanced hepatocellular carcinoma [4,5,6]. However, adverse side effects on patient’s health and a high-dose drug burden with increased treatment costs necessitate the development of more effective treatment options [7,8]. Therefore, it is desirable to identify natural compounds with minimal detrimental effects as alternative therapeutic agents in liver cancer treatment.

Apoptosis is the preferred cell death in cancer treatment due to its ability to completely dismantle the cell from within in an organized manner without affecting neighbouring cells [9]. The process of apoptosis involves cell shrinkage, membrane alteration, exposure of phosphatidylserine (PS) to the outer plasma membrane, chromatin cleavage, and oligonucleosomal deoxyribonucleic acid (DNA) fragmentation [10,11]. On the other hand, necrosis is a cell death process that affects neighbouring cells and is characterized by attributes such as cell swelling, loss in plasma membrane integrity, and random DNA digestion [11,12]. 

Terpene is the largest class of compounds found abundantly rich in plants and famous for its medicinal values [13]. Pentacyclic triterpenes have the most potent anti-cancer properties, and both the natural and synthetically-derived compounds have been investigated as chemopreventive and chemotherapeutic agents, with some having minimal adverse effects on normal cells [14,15,16].

**Figure 1:**
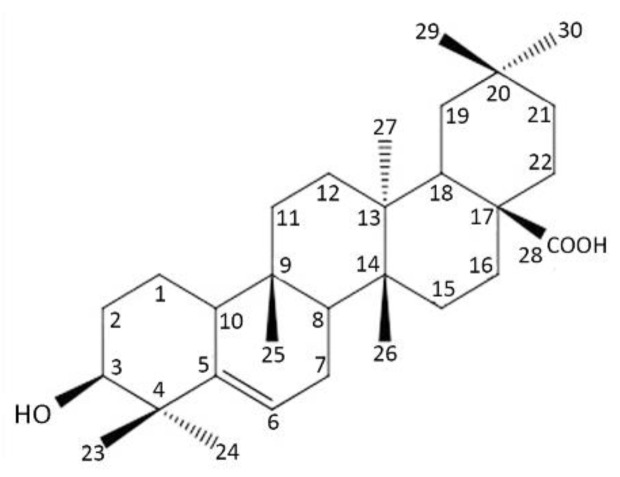
Structure of pinnatane A.

*Walsura pinnata* also natively known in Malaysia as “*lantupak mata kucing*,” is a mahogany tree that belongs to the Meliaceae family and is distributed across Asia from Yunnan, China, towards West Papua, New Guinea [17]. The rounded canopy tree is 12 m to 37 m tall and is populates lowland rainforests [18]. Some triterpenes that have been isolated from dichloromethane extract of *W. pinnata* showed moderate cytotoxicity towards human breast cancer cells (MCF-7), human ovarian cancer cells (SK-OV-3) [19], and a good effect against leukaemia stem cells [20]. In a previous study of the hexane extract of *W. pinnata* bark, a cytotoxic effect against a human liver cancer (HepG2) cell line was seen at 50.00% inhibitory concentration (IC_50_) value of 5.0 µg/mL [21]. In this study, pinnatane A (Figure 1), a rare glutinane type triterpenoid isolated from the hexane crude of *W. pinnata* bark, was investigated for its potential cytotoxic ability against cancer cells.

## 2. Results

### 2.1. Structure of Pinnatane A

Pinnatane A was obtained as a white crystal (melting point = 306 °C); ${\left[\alpha \right]}_{D}^{25}$ +54 (c = 0.01, MeOH); ESIMS (*m*/*z*) 455.3531 (M − H)^−^. Pinnatane A, structurally identified as 3β-hydroxy-5-glutinen-28-oic acid, is a glutinane type of pentacyclic triterpenoid with significant functional attachments of a hydroxyl group at C-3, double bond at C-5(6) and carboxyl group at C-17. Pinnatane A was characterized from spectral data (Table S1; Figures S1–S8) based on previous studies [19,22,23]. Pinnatane A was dissolved in dimethyl sulfoxide (DMSO) prior to biological activity assays.

### 2.2. Pinnatane A Induced Cytotoxic Effects in Cancer Cell Lines

The cytotoxic activity of pinnatane A was determined using the 3-(4,5-Dimethyl-2-thiazolyl)-2,5-diphenyl-2H-tetrazolium bromide (MTT) assay by measuring the metabolic activity in viable cells. The results demonstrated that pinnatane A is cytotoxic in a dose-dependent manner on various cancer cell lines after 24 h. All cell lines showed a decreasing percentage of viable cells when exposed to increasing concentration of pinnatane A, with different cytotoxic selectivity in the tested cell lines Table 1.

Normal human lung fibroblast cell line, MRC-5, was used to compare the selective cytotoxic activity of pinnatane A in cancer cells. The IC_50_ value of MRC-5 cells was identified to be 48.8 ± 1.0 µM. Pinnatane A has the lowest IC_50_ value against the Hep3B cell line, with the value of 19.0 ± 0.5 µM, followed by two human bladder cancer cell lines, EJ-28 and RT-112, with IC_50_ values of 33.9 ± 3.8 µM and 48.0 ± 4.6 µM, respectively. The IC_50_ value of MRC-5 cells was compared with all tested cancer cell lines to calculate the selectivity index (SI) values. SI value is the degree of selectivity of the compound in inducing cytotoxicity in cancer cells without inducing toxicity in normal cells. The larger the SI value, the more selective it is [24]. Among all the cancer cells tested, only one cell line had an SI value higher than 2, which was Hep3B cell line with value of 2.57. Thus, the Hep3B cell line was selected as the main cell line for further analysis, and the HepG2 cell line was used to compare the cytotoxic effects of pinnatane A between two different liver cancer cell lines.

The MTT assay was repeated for a time-dependent study for Hep3B, HepG2, and MRC-5 cell lines treated with pinnatane A under various incubation times (12, 48, and 72 h). The results showed that cytotoxic activity was time-dependent in both Hep3B and HepG2 cell lines (Table 2). In contrast, the IC_50_ values of the MRC-5 cell line remained in the same range between 40.0 µM to 60.0 µM at 24, 48, and 72 h of treatments, which showed no significant difference in cytotoxic activity for time points longer than 24 h in the MRC-5 cell line. The IC_50_ values of treated Hep3B and HepG2 cell lines at 48 h were 13.5 ± 1.6 µM and 17.1 ± 2.1 µM, respectively. The SI values calculated for both liver cancer cell lines in comparison with the MRC-5 cell line at 48 h exceeded 3 for both Hep3B (SI = 3.95) and HepG2 (SI = 3.12) cells, which are regarded to be good selectivity indices [25].

### 2.3. Pinnatane A Triggered Membrane Integrity Disruption in Liver Cancer Cells

**Figure 2:**
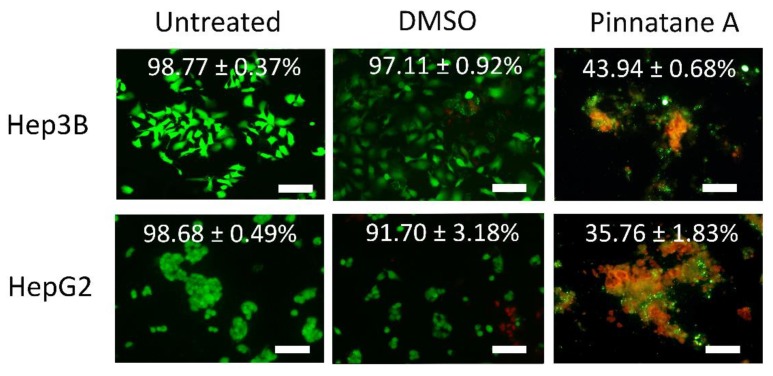
Pinnatane A induces cell death in Hep3B and HepG2 cell lines. A live/dead assay after treatment with pinnatane A and DMSO for 12 h. Green fluorescence denotes viable cells stained with calcein-AM, while reddish-orange fluorescence represents dead cells stained with ethidium homodimer. All results are expressed as a total percentage of viable cells from four random fields with mean ± standard deviation (SD) of three independent determinations. Scale bar represents 100 μm.

A live/dead assay was performed to confirm the cytotoxic activity of pinnatane A towards liver cancer cell lines. These cells were treated with pinnatane A at a high dose based on the IC_50_ value for 12 h (Table 2). Then, dual staining with fluorescence dyes was performed using calcein-AM and an ethidium homodimer. The results showed that the percentage of viable cells decreased for Hep3B cells from 98.77 ± 0.37% to 43.94 ± 0.68% and HepG2 cells from 98.68 ± 0.49% to 35.76 ± 1.83% (Figure 2). Both cell lines showed a significant decrease in viable cells after being treated with pinnatane A for 12 h compared to DMSO-treated and untreated controls. These results suggest that pinnatane A is a potential cytotoxic agent for both liver cancer cell lines.

### 2.4. Pinnatane A Encouraged Cell Cycle Arrest in Liver Cancer Cells

**Figure 3:**
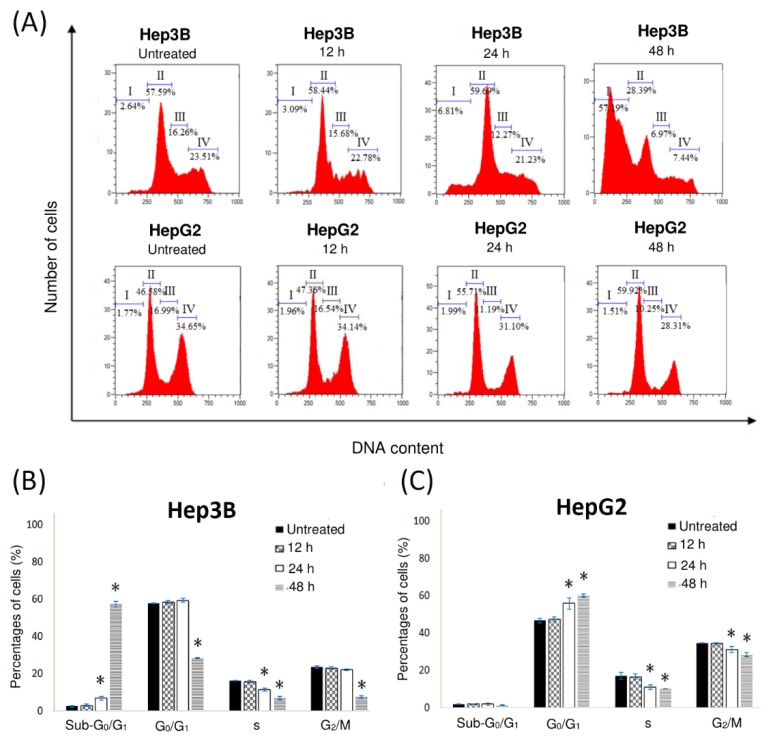
Pinnatane A caused cell cycle arrest in Hep3B and HepG2 cell lines. (**A**) Cell cycle distribution of Hep3B and HepG2 cells with pinnatane A treatment for various time points (12, 24, and 48 h) using flow cytometry after staining with PI. (**B**) Hep3B and (**C**) HepG2 cells cell cycle phase distribution presented in four groups, which are the group I: sub-G_0_/G_1_ phase, group II: G_0_/G_1_ phase, group III: S phase, and group IV: G_2_/M phase. All results are expressed in the histogram as total percentages of cells from four different groups with mean ± SD of three independent determinations. All data collected from experiments were performed in three replicates and analyzed using the one-way analysis of variance (ANOVA) at a significance level of *p* < 0.05 and indicated by *.

Cell cycle analysis was used to demonstrate the influence of pinnatane A toward the growth of liver cancer cells. The cell lines were treated for 12, 24, and 48 h before fixing and staining with propidium iodide (PI) to examine the DNA contents using flow cytometry. Cell cycle analysis was carried out to classify the cell population into distinct phases, which were the sub-G_0_/G_1_ phase (group I), G_0_/G_1_ phase (group II), S phase (group III), and G_2_/M phase (group IV) in Figure 3A. Treatment of pinnatane A on Hep3B cells after 48 h showed an increase of population in the sub-G_0_/G_1_ phase from 2.64 ± 0.33% to 57.19 ± 1.50%. A significant decrease in the S phase from 16.26 ± 0.24% to 6.97 ± 1.00% and G_2_/M phase from 23.51 ± 0.65% to 7.44 ± 0.72% after 48 h treatment supported the G_0_/G_1_ cell cycle arrest result (Figure 3B). On the other hand, the HepG2 cell population increased the G_0_/G_1_ phase from 46.58 ± 1.28% to 59.92 ± 0.95% without any significant changes in the sub-G_0_/G_1_ phase, suggesting no induction of apoptosis (Figure 3C). A significant decrease in S phase from 16.99 ± 1.29% to 10.25 ± 0.95% and G_2_/M phase from 34.65 ± 0.20% to 28.31 ± 0.94% concluded the cell arrest of the HepG2 cell line in the G_0_/G_1_ phase.

### 2.5. Pinnatane A Initiated Apoptosis and Necrosis in Liver Cancer Cells

**Figure 4:**
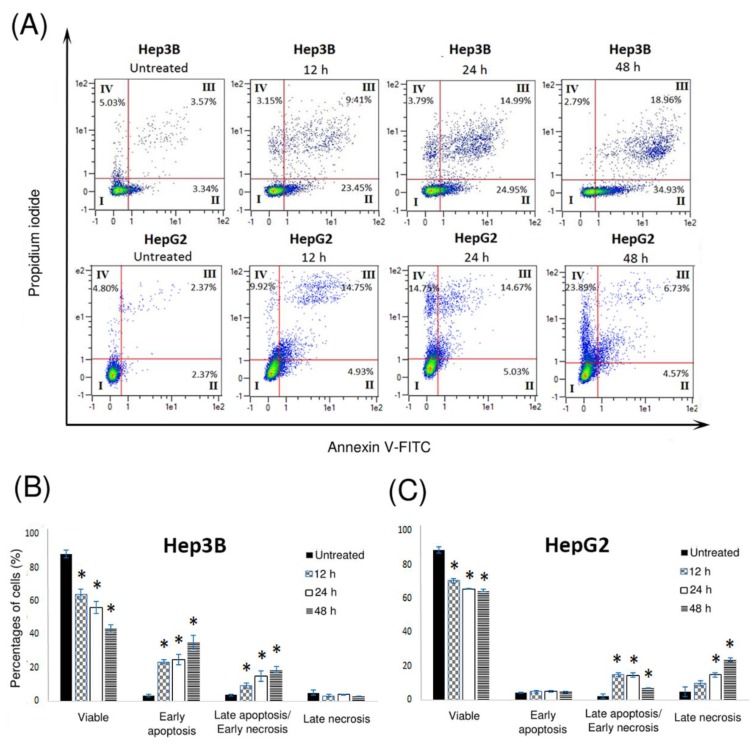
Pinnatane A induced apoptosis in Hep3B and necrosis in HepG2 cells. (**A**) Detection of apoptosis and necrosis using annexin V-FITC and PI dual staining on Hep3B and HepG2 cell lines treated with pinnatane A at 12, 24, and 48 h. (**B**) Hep3B and (**C**) HepG2 cell lines population were distributed as follows: I: non-stained cells indicating viable cells, II: annexin V-FITC stained indicating early apoptosis, III: annexin V-FITC and PI stained cells indicating late apoptosis or early necrosis, and IV: PI stained cells indicating late necrosis. All results are expressed in the histogram as total percentages of cells from four different quadrants with mean ± SD of three independent determinations. All data collected from experiments were performed in three replicates and analyzed using the one-way analysis of variance (ANOVA) at a significance level of *p* < 0.05 and indicated by *.

The distribution of cells undergoing apoptosis or necrosis was analyzed using annexin V-fluorescein isothiocyanate (annexin V-FITC/PI) flow cytometry assay in Hep3B and HepG2 cells treated with pinnatane A for 12, 24, and 48 h. The stages of cell death were presented in four different quadrants (Figure 4A). Cells that are undergoing apoptosis will shift from the viable quadrant (I) to the early apoptosis quadrant (II), and eventually end up in late apoptosis quadrant (III). On the other hand, cells that undergo necrosis will shift from viable quadrant (I) to the late necrosis quadrant (IV). Pinnatane A induced apoptosis in Hep3B cells by significantly increasing the population of cells undergoing early apoptosis from 3.34 ± 0.79% to 34.93 ± 4.46% and late apoptosis from 3.58 ± 0.40% to 18.96 ± 1.91% after 48 h of treatment with no significant changes in the necrotic population (Figure 4B). In the HepG2 cell line, the cell population in late necrosis increased significantly from 4.80 ± 1.84% to 23.89 ± 1.02% (Figure 4C). Thus, these findings suggest that pinnatane A induced apoptosis in Hep3B and necrosis in HepG2 cell lines.

### 2.6. Pinnatane A Caused Different DNA Degradation Patterns

**Figure 5:**
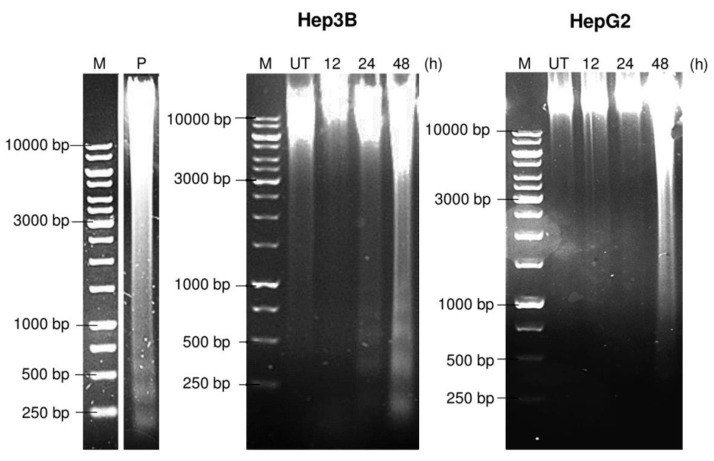
Pinnatane A induced DNA laddering on Hep3B and smearing on HepG2 cells. A DNA fragmentation assay using agarose gel electrophoresis for Hep3B and HepG2 cells treated with pinnatane A at 12, 24, and 48 h. M: marker; UT: Untreated. P: Positive control Hep3B cells treated with cisplatin.

In order to validate the mode of cell death induced by pinnatane A, treatment for 12, 24, and 48 h was carried out in both Hep3B and HepG2 cell lines, where agarose gel electrophoresis of DNA was performed. After 48 h of treatment, a laddering pattern of genomic DNA was observed in the Hep3B cell line, while a smear pattern was observed in the HepG2 cell line (Figure 5). One of the major hallmarks of apoptosis is oligonucleosomal DNA degradation at the late stage of apoptosis. Chromatin DNA in apoptotic cells breaks at the junction between nucleosomal units visualized as a laddering pattern in electrophoresis. In contrast, the DNA of cells that undergo necrosis will appear as smears due to the random degradation of DNA [11]. However, this has to be further validated with other assays such as terminal deoxynucleotidyl transferase dUTP nick end labelling (TUNEL).

## 3. Discussion

Natural products have played an immense role in the treatment of countless diseases, notably cancer and bacterial infections. Triterpenes became the highlight in anti-cancer drug testing due to its cytotoxic effects on cancer cell lines with minimal effects on normal cells, such as betulinic acid and oleanolic acid [26,27]. Initial cytotoxicity study of betulinic acid identified its selective cytotoxic ability against melanoma [28], and further investigation identified multiple cancer types such as lung, ovarian, and cervical cancers [14]. In addition, the ability of oleanolic acid in inhibiting multi-drug resistance was suggested to be beneficial in cancer patients undergoing chemotherapy [29,30]. 

In this study, glutinane type triterpenoid, pinnatane A, was used to investigate its cytotoxic activity, cell arrest effects, and cell death mechanism induced in human liver cancer cells. 

An MTT assay showed dose- and time-dependent cytotoxic activities. However, different cell types have different sensitivity towards pinnatane A. Among the cancer cell lines tested at 24 h, the liver Hep3B cell line was the only cell line that had an IC_50_ value below 25.0 µM, the recommended biological activity assay endpoint for a pure compound [31]. Pinnatane A was seen as effective towards Hep3B cells with an IC_50_ value of less than 4.0 µg/mL (equivalent to 8.8 µM) after 72 h of treatment [32,33]. This study revealed that pinnatane A was able to induce cell death in cancer cells with minimal cytotoxic effects on normal cells.

A live/dead assay enabled microscopic visualization of viable and dead cells. Fluorescence viability stains enabled the visualization of cell appearance based on membrane integrity, which were not observed in Hep3B and HepG2 cells after being treated with pinnatane A. 

Cell cycle analysis was conducted in order to identify the induction of cell cycle arrest. In Hep3B cells, treatment with pinnatane A significantly increased the cell population in the sub-G_0_/G_1_ phase due to the formation of hypodiploid DNA in apoptotic bodies of cells [34]. In contrast, HepG2 cells remain in G_0_/G_1_ phase. The diverse effects shown by pinnatane A treatment in HepG2 and Hep3B cell lines were also observed in a previous study with another triterpene, ganoderiol F, where treatment in HepG2 cells induced G_1_ phase arrest but not in Hep3B cells [35]. Compounds that enable the interruption of cell cycle progression will help in controlling tumour growth and eventual killing of the cancer cells [36].

The outer membrane protrusion of PS is important in keeping the homeostasis of the human body by signalling phagocytes to engulf dead cells, which is the preferred death mechanism in cancer treatment [37]. Apoptotic cells are characterized by a lag period between annexin V-FITC positivity and PI positivity, but in necrotic cells both events are seen to coincide [38]. In the present study, pinnatane A induced different modes of cell death, which are apoptosis in Hep3B and necrosis in HepG2 cell lines. These findings are in contrast with triterpenoid oleanolic acid and ursolic acid that are capable of inducing caspase-dependent apoptosis and triggering DNA fragmentation in both the Hep3B and HepG2 cell lines [39]. However, triterpenoid asiatic acid was reported to predominantly trigger necrosis in glioblastoma cells but induces apoptosis in colon cancer, which highlight the potential of triterpenoid in inducing different mechanisms of cell death in different cancer types [40].

Pinnatane A was found to induce DNA fragmentation in Hep3B cells. An apoptotic DNA laddering pattern was also reported in human CEM lymphocytes induced by saikosaponin, a triterpene saponin associated with *c-myc*, *p53* and *bcl-2* mRNA [41]. On the other hand, DNA degradation in HepG2 may have alternative death pathways. 

Thus, in this preliminary study, cytotoxicity effect of pinnatane A on the two liver cancer cell lines was determined using MTT and live/dead assays, while apoptosis was identified using annexin V-FITC/PI and DNA fragmentation. The cell cycle analysis showed the ability of pinnatane A to halt growth at G_0_/G_1_ phase.

## 4. Materials and Methods 

### 4.1. Reagent and Chemicals

Dichloromethane (DCM), n-hexane, ethyl acetate (EtOAc), deuterated chloroform (CDCl_3_) (deuteration degree minimum 99.80%), silica gel (Merck 60, 230–400 mesh, Merck, Darmstadt, Germany) and thin layer chromatography (TLC) (Merck 60 GF254, Merck, Germany) were used for extraction, isolation, and characterization of pinnatane A. Each solvent was of analytical grade and distilled before use. The culture media and supplements listed below were purchased as stated: foetal bovine serum (FBS) and sodium pyruvate (Sigma-Aldrich, St. Louis, MO, USA), Dulbecco modified Eagle medium (DMEM) supplemented with 4.5 g/L glucose and 300.0 mg/L l-glutamine (Hyclone Laboratories, South Logan, UT, USA), Roswell Park Memorial Institute 1640 medium (RPMI 1640) (Thermo Fisher Scientific, Waltham, MA, USA), Minimum Essential Medium Eagle (EMEM) (Sigma-Aldrich, USA), and Minimum Essential Medium Alpha (MEM-α) (Nacalai Tesque, Kyoto, Japan). The reagents and kits listed below were obtained as stated: ethanol (Merck), dimethyl sulfoxide (DMSO) (Fisher Scientific), cisplatin and 3-(4,5-dimethyl-2-thiazolyl)-2,5-diphenyl-2H-tetrazolium bromide (MTT) reagent (EMD Chemicals, Gibbstown, NJ. USA), and FITC Annexin V Apoptosis Detection Kit I BD Pharmingen^TM^ (Becton, Dickinson & Co, Franklin Lakes, NJ, USA), ribonuclease A (RNase A), and propidium iodide (PI) (Nacalai Tesque), LIVE/DEAD^®^ Viability/Cytotoxicity kit for mammalian cells, ApoTarget^TM^ Quick Apoptotic DNA Ladder Detection Kit (Invitrogen, Carlsbad, CA, USA), and RedSafe Nucleic Acid Staining solution (iNtRON Biotechnology, Gyeonggi-Do, South Korea).

### 4.2. Plant Materials

The plant materials of *W. pinnata* were collected from a rainforest over 243 km from Gua Musang, Kelantan to Kuala Lipis, Pahang in Malaysian peninsular forest. The sample was identified by Tarelli. O. and deposited in the Herbarium of the Chemistry Department, Faculty of Science, University of Malaya, Malaysia with the voucher specimen number KL 4571.

### 4.3. Extraction, Isolation, and Characterization of Pinnatane A

Air-dried and powdered bark of *W. pinnata* (2.3 kg) was de-fatted with n-hexane for 72 h using a simple maceration method. Periodical stirring was applied throughout the duration to enhance the extraction yield. After three days, the solvent was filtered through a filter paper and was concentrated at 40 °C using a rotary vacuum evaporator (Rotavapor R-114, BÜCHI, Flawil, Switzerland) to obtain a dark-brown gummy crude extract (25.0 g). The hexane crude extract (10.0 g) was subjected to open column chromatography (CC) using n-hexane, n-hexane:EtOAc, and EtOAc:MeOH gradually to yield 24 major fractions. The twenty-fourth fraction, eluted with n-hexane:EtOAc, gave 2.0 g of the product, which was further purified using CC (n-hexane:acetone, 94:6) to yield pinnatane A in the form of a white powder crystal (5.0 mg). Observation of fraction separation was done using TLC with silica gel 60 GF254 plates and identified using vanillin reagent. All spectral data were obtained on the following instruments: the 1-D and 2-D NMR were recorded in CDCl_3_ using BRUKER Avance II 400 MHz (Bruker Analytische GmbH, Billerica, MA, USA) and CDCl_3_ peak (^1^H, 7.26 ppm; ^13^C, 77.00 ppm) was used as a reference peak. The mass spectra were obtained on a Agilent 6530 Accurate-Mass Q-TOF ESI liquid chromatography-mass spectrometry (LC/MS) (Agilent Technologies, Santa Clara, CA, USA) and the IR spectra were obtained on a FT-IR spectrometer RX1 (Perkin-Elmer, Waltham, MA, USA). The structure of pinnatane A was determined based on the comparison of ^1^H and ^13^C Nuclear Magnetic Resonance (NMR) spectroscopy data reported in the literature [23].

### 4.4. Cultivation of Cell Lines

A total of six pairs of human cancer cell lines were used in this study: bladder (EJ-28 and RT-112), breast (MCF-7 and MDA-MB-231), cervical (HeLa S3 and SiHa), liver (HepG2 and Hep3B), lung (A549 and SK-LU-1), and prostate (PC-3 and DU 145). Human lung fibroblast (MRC-5) cell line was used as the normal cell line control. All cell lines were obtained from American Type Culture Collection, USA, except for MDA-MB-231 and SK-LU-1 (AseaCyte, Selangor, Malaysia). Each cell line was maintained in an appropriate culture medium: the HeLa S3, HepG2, and SiHa cell lines were cultured in DMEM; the Hep3B and MRC-5 cell lines were cultured in EMEM supplemented with 1.00% (*v/v*) sodium pyruvate; the SK-LU-1 cell line was cultured in MEM-α; while the A549, DU 145, EJ-28, MCF-7, MDA-MB-231, PC-3, and RT-112 were cultured in RPMI-1640. All cultured media were supplemented with 10.00% (*v/v*) FBS. All cell lines were allowed to grow as monolayers and maintained in an incubator at 37 °C, 5.00% CO_2_, and 95.00% humidified air. 

### 4.5. MTT Assay

All cell lines were seeded a total of 1.0 × 10^4^ cells/well in 96-well plate and incubated overnight before being treated with pinnatane A at various concentrations (0–100.0 µM) and incubated for another 24 h. After incubation, 20.0 µL of MTT reagent (5.0 mg/mL) was added to each well, followed by incubation in the dark at 37 °C for 90 min. Spent media were discarded and purple formazan precipitates were dissolved in 200.0 µL DMSO. Results were obtained using a microtiter plate reader (Tecan Sunrise^TM^, Männedorf, Switzerland) at a test wavelength of 570 nm and a reference wavelength of 650 nm to detect the absorbance of the solution. From the readings, percentages of viable cells were calculated with respect to the DMSO control and a 50.00% inhibitory concentration (IC_50_) was indicated from the dose-response curve fitting graph at a 50.00% viability of cells. The steps were repeated only for Hep3B, HepG2, and MRC-5 cells at 12, 48, and 72 h. The selectivity index (SI) values were determined to identify the relative effectiveness of pinnatane A in inducing cancer cells death compared to normal cells death and were calculated as follows:

SI = IC_50_ value of normal cells/IC_50_ value of cancer cells (1)

### 4.6. Live/Dead Assay

Qualitative assessment of cell viability upon treatment with pinnatane A was conducted using the LIVE/DEAD^®^ Viability/Cytotoxicity Kit. A total of 2.0 × 10^5^ cells/well were plated on the surface of a sterile glass coverslip placed in a six-well plate and incubated overnight before treatment at IC_50_ value for 12 h to avoid an inaccurate result. Untreated and DMSO-treated cells were used as controls. Spent media were aspirated and cells were washed with 1× PBS solution before staining. Cells were stained using a dual-fluorescence system of 150.0 µL of calcein-AM (2.0 µM) and ethidium homodimer (4.0 µM). Excitation and emission wavelengths were set at 494/517 nm for calcein-AM, while 528/617 nm for ethidium homodimer visualized as green (viable cells) and red (dead cells) fluorescence, respectively. Visualization of samples was carried out using a Nikon Eclipse TS-100 fluorescence microscope (Nikon, Tokyo, Japan) under 100× magnification. Four random fields of view for each sample were captured and the percentages of viable cells were calculated as follows:

Viable cells (%) = [live cells/(live cells + dead cells)] × 100(2)

### 4.7. Cell Cycle Analysis

A total of 1.0 × 10^6^ cells were treated with pinnatane A at IC_50_ value for 48 h and incubated for 12, 24, and 48 h, then washed twice with 1× PBS solution. The cell pellets were re-suspended in 1.0 mL of 1× PBS solution and 3.0 mL of 70.00% ethanol before overnight fixation at 4 °C. The cells were then washed twice with 1× PBS solution at a high centrifugation speed. The cell pellets were stained with 500.0 µL of PI solution (50.00 µg/mL), 5.0 µL RNase A (10.0 mg/mL), and incubated in the dark for 45 min. The samples were analysed using MACSQuant^®^ Analyzer 10 flow cytometry with MACSQuantify™ version 2.10 software (Miltenyi Biotec, Bergisch Gladbach, Germany). All results were expressed in a histogram as a total percentages of cells from four different cell cycle phases.

### 4.8. Annexin V-FITC/PI Flow Cytometry Assay

Apoptosis was measured using a FITC Annexin V Apoptosis Detection Kit I according to the manufacturer’s instruction. A total of 5.0 × 10^5^ cells were cultured before being treated with pinnatane A at the IC_50_ value for 48 h and incubated for 12, 24, and 48 h. Cells were harvested from both floating and attached cells and washed twice using 1× PBS solution and further incubated for 15 min in the dark with 100.0 µL of binding buffer containing 5.0 µL of annexin V-FITC and PI respectively. The samples were mixed with 400.0 µL binding buffer before being analyzed using MACSQuant^®^ Analyzer 10 flow cytometry with MACSQuantify™ version 2.10 software (Miltenyi Biotec). All results were expressed in a scatter plot as total percentages of cell population from four different quadrants representing different stages of cell death.

### 4.9. DNA Fragmentation Assay

Cells were cultured until 80.00% confluency before being treated with pinnatane A at the IC_50_ value for 48 h and incubated for 12, 24, and 48 h. Hep3B cells treated with cisplatin were used as a positive control for apoptotic DNA laddering. The cells were harvested and extracted using ApoTarget™ Quick Apoptotic DNA Ladder Detection according to the manufacturer’s protocol. DNA extracts were analyzed in 1.00% agarose gel mixed with RedSafe Nucleic Acid Staining solution through electrophoresis. Fragmentation of DNA was observed under ultraviolet illumination and visualized using a Fusion FX7-7027 (Vilber, Eberhardzell, Germany) gel documentation system.

### 4.10. Statistical Analysis

Results were expressed as mean values ± standard deviation (SD). All data collected from experiments were performed in three replicates and analyzed using the one-way analysis of variance (ANOVA) at a significance level of *p* < 0.05 and indicated by *.

## 5. Conclusions

This study investigated the properties of pinnatane A from *W. pinnata* in inducing cytotoxic activities in Hep3B and HepG2 cell lines, which include cytotoxicity and cell cycle analysis assays. Treatment with pinnatane A resulted in G_0_/G_1_ phase cell cycle arrest in both Hep3B and HepG2 cell lines, and was also found to induce two different types of cell death, which were apoptosis in Hep3B and necrosis in HepG2 cell lines. This study has shown the potential of a glutinane triterpenoid, pinnatane A, to induce effective cytotoxicity in liver cancer cell lines. Further studies are needed to fully understand and validate the mechanism of cell death in Hep3B and HepG2 cell lines and its relevant regulatory pathways.

## Figures and Tables

**Table 1:** The effects of pinnatane A treatment for 24 h on various cell lines.

Human Cell Lines	IC_50_ (µM)	SI ^a^
Normal lung fibroblast (MRC-5)	48.8 ± 1.0	N.A. ^c^
Breast adenocarcinoma (MCF-7)	60.9 ± 2.3	0.80
Breast adenocarcinoma (MDA-MB-231)	92.9 ± 3.1	0.53
Bladder carcinoma (EJ-28)	33.9 ± 3.8	1.44
Bladder carcinoma (RT-112)	48.0 ± 4.6	1.02
Cervical adenocarcinoma (HeLa S3)	59.7 ± 0.9	0.82
Cervical carcinoma (SiHa)	>100 ^b^	N.C. ^d^
Hepatocellular carcinoma (Hep3B)	19.0 ± 0.5	2.57
Hepatocellular carcinoma (HepG2)	55.8 ± 2.3	0.87
Lung adenocarcinoma (A549)	50.9 ± 3.1	0.96
Lung adenocarcinoma (SK-LU-1)	>100 ^b^	N.C. ^d^
Prostate carcinoma (DU 145)	87.2 ± 2.1	0.56
Prostate adenocarcinoma (PC-3)	55.3 ± 3.9	0.88

^a^ SI = IC_50_ value of normal cells/IC_50_ value of cancer cells; ^b^ denotes an overall cell viability level of > 50.00% after treatment with pinnatane A at 100.0 μM for 24 h; ^c^ N.A.: Not applicable; ^d^ N.C.: Not calculated because IC_50_ value was not determined.

**Table 2:** The time-dependent effects of pinnatane A on liver cancer cell lines.

Time (h)	MRC-5	Hep3B		HepG2	
	IC_50_ (µM)	IC_50_ (µM)	SI ^a^	IC_50_ (µM)	SI ^a^
12	86.0 ± 4.5	52.7 ± 5.5	1.63	70.7 ± 0.5	1.22
24	48.6 ± 1.2	19.0 ± 0.5	2.56	55.8 ± 2.3	0.87
48	53.3 ± 4.6	13.5 ± 1.6	3.95	17.1 ± 2.1	3.12
72	53.1 ± 0.8	5.0 ± 0.0	10.62	8.8 ± 0.6	6.03

^a^ SI = IC_50_ value of normal cells/IC_50_ value of cancer cells.

## Supplementary Materials

The following are available online: Table S1: ^1^H (600 MHz) and ^13^C (150 MHz) Nuclear Magnetic Resonance (NMR) data of pinnatane A in CDCl_3_. Figure S1: ^1^H (600 MHz) NMR spectrum of pinnatane A. Figure S2: ^13^C (150 MHz) NMR spectrum of pinnatane A. Figure S3: Distortionless enhancement by polarization transfer-135 (DEPT-135) spectrum of pinnatane A. Figure S4: Homonuclear correlation spectroscopy (COSY) spectrum of pinnatane A. Figure S5: Heteronuclear multiple bond correlation (HMBC) spectrum of pinnatane A. Figure S6: Selected COSY and HMBC correlations of pinnatane A. Figure S7: Infrared (IR) spectrum of pinnatane A. Figure S8: Liquid chromatography–mass spectrometry (LC-MS) spectrum of pinnatane A.
