# Supplementary material for: Cytotoxic Effects of Pinnatane A Extracted from Walsura pinnata (Meliaceae) on Human Liver Cancer Cells
Source: Molecules. 2018 Oct 23;23(11):2733. doi: 10.3390/molecules23112733 (PMC6278294; doi:10.3390/molecules23112733)
Supplement: Supplementary file 1 [file molecules-23-02733-s001.pdf]

Supplementary Materials

Cytotoxic Effects of Pinnatane A Extracted from *Walsura pinnata* (Meliaceae) on Human Liver Cancer Cells

Nurhisyam Zakaria [1]; Mohamad Azrul Mahdzir [2], Mahfuzah Yusoff [3], Norhafiza Mohd Arshad [4], Khalijah Awang [2, 5]; Noor Hasima Nagoor [1, 4, *]

[1] Institute of Biological Sciences, Faculty of Science, University of Malaya, 50603 Kuala Lumpur Malaysia; nurhisyam2309@gmail.com

[2] Department of Chemistry, Faculty of Science, University of Malaya, 50603 Kuala Lumpur Malaysia; mazrulm89@gmail.com (M.A.M); khalijah@um.edu.my (K.A.)

[3] Centre for Foundation Studies in Science, University of Malaya, 50603 Kuala Lumpur, Malaysia; mahfuzahyusoff@um.edu.my

[4] Centre for Research in Biotechnology for Agriculture (CEBAR), University of Malaya, 50603 Kuala Lumpur Malaysia; hafizaarshad@um.edu.my

[5] Centre of Natural Products and Drug Discovery (CENAR), University of Malaya, 50603 Kuala Lumpur, Malaysia.

* Correspondence: hasima@um.edu.my; Tel.: +603-7967-5921

Content: **Page**

Table S1: ^{1}H (600 MHz) and ^{13}C (150 MHz) Nuclear Magnetic Resonance (NMR) data of pinnatane A in $CDCl_3$. 2
Figure S1: ^{1}H (600 MHz) NMR spectrum of pinnatane A. 3
Figure S2: ^{13}C (150 MHz) NMR spectrum of pinnatane A. 3
Figure S3: Distortionless enhancement by polarization transfer -135 (DEPT-135) spectrum of pinnatane A. 4
Figure S4: Homonuclear correlation spectroscopy (COSY) spectrum of pinnatane A. 4
Figure S5: Heteronuclear multiple bond correlation (HMBC) spectrum of pinnatane A. 5
Figure S6: Selected COSY and HMBC correlations of pinnatane A. 5
Figure S7: Infrared (IR) spectrum of pinnatane A. 6
Figure S8: Liquid chromatography–mass spectrometry (LC-MS) spectrum of pinnatane A. 7
Related references 7

Table S1: ^1H (600 MHz) and ^{13}C (150 MHz) Nuclear Magnetic Resonance (NMR) data of pinnatane A in $CDCl_3$.

Position	Pinnatane A		3*β*-hydroxy-5-glutinen-28-oic acid (Elfita *et al.*, 2009)	
	δ_H (ppm), *J* (Hz)	δ_C (ppm)	δ_H (ppm), *J* (Hz)	δ_C (ppm)
1	1.45, *m*<br>1.50, *m*	18.3	1.44<br>1.52	18.3
2	1.67, *m*<br>1.85, *m*	27.8	1.67<br>1.82	27.8
3	3.47, *dd* (3.2, 2.3)	76.3	3.45, *br s*	76.3
4	-	40.8	-	40.8
5	-	141.6	-	141.5
6	5.64, *d* (5.8)	121.7	5.6	121.7
7	1.77, *m*<br>1.95, *m*	23.5	1.75<br>1.95	23.4
8	1.52, *m*	47.7	1.5	47.7
9	-	35.1	-	35.1
10	2.00, *m*	49.4	1.98	49.9
11	1.36, *m*<br>1.53	34.5	1.36<br>1.51	34.5
12	1.39, *m*<br>1.47, *m*	30.9	1.35<br>1.45	30.9
13	-	38.7	-	38.6
14	-	37.2	-	37.1
15	1.21, *m*<br>1.25, *m*	32.5	1.2	32.4
16	1.50, *m*	35.8	1.48	35.8
17	-	44.7	-	44.7
18	2.43, *dd* (13.2, 4.5)	37.8	2.37, *dd* (13.1, 3.8)	37.7
19	1.17, *m*<br>1.31, *m*	34.9	1.14<br>1.29	34.8
20	-	28.5	-	28.5
21	1.47, *m*	32.8	1.46	32.8
22	1.67, *m*<br>2.29, *dd* (14.9, 9.7)	29.4	1.65<br>2.29, *dd* (14.7, 9.5)	29.5
23	1.04, *s*	28.9	1.01, *s*	28.9
24	1.14, *s*	25.4	1.12, *s*	25.4
25	0.82, *s*	15.6	0.80, *s*	15.6
26	0.93, *s*	20.3	0.89, *s*	20.3
27	1.04, *s*	18.2	0.96, *s*	18.2
28	-	182.6	-	184.8
29	0.98, *s*	34.3	0.91, *s*	34.3
30	0.94, *s*	29.8	1.01, *s*	29.7

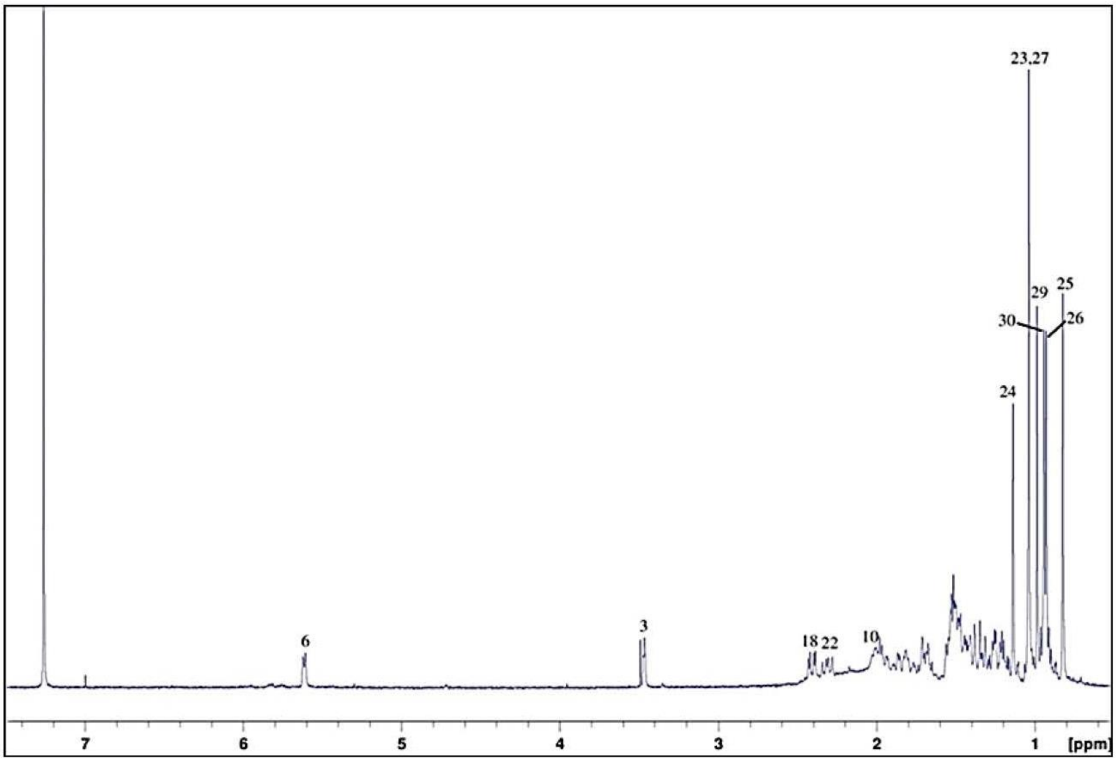

Figure S1: ^{1}H (600 MHz) NMR spectrum of pinnatane A.

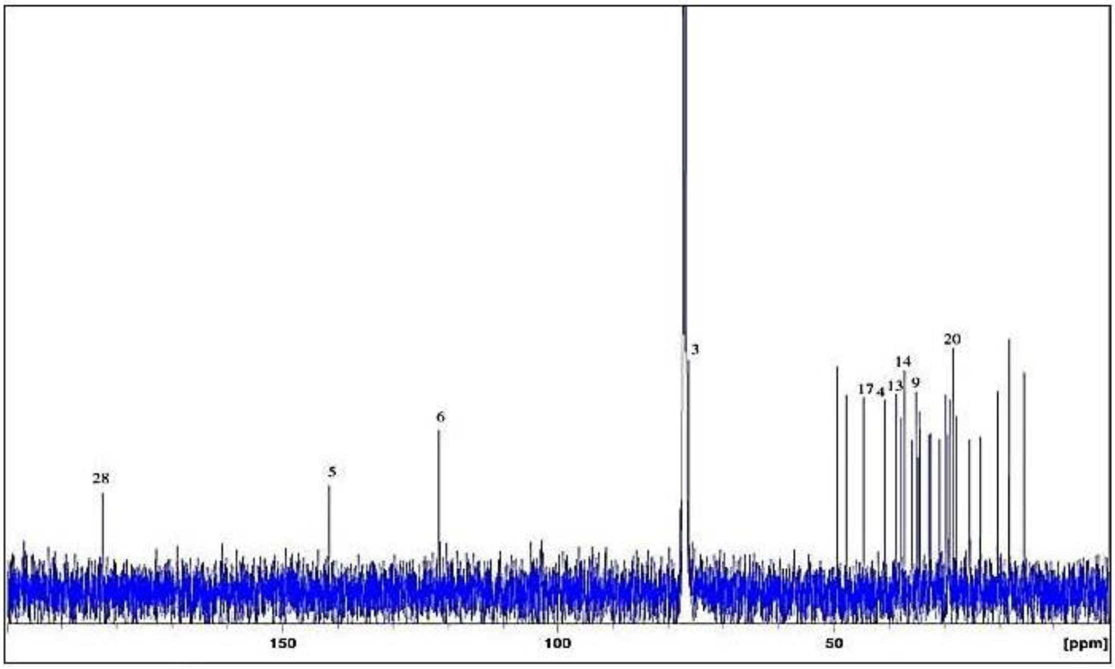

Figure S2: ^{13}C (150 MHz) NMR spectrum of pinnatane A.

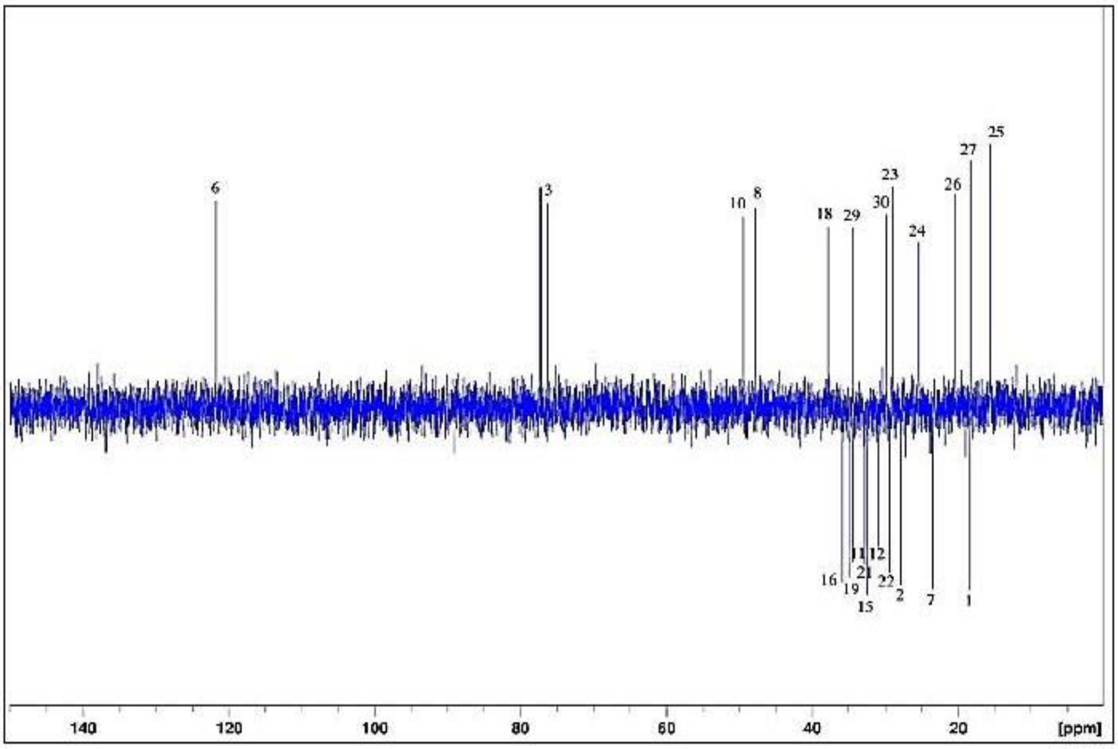

Figure S3: Distortionless enhancement by polarization transfer -135 (DEPT-135) spectrum of pinnatane A.

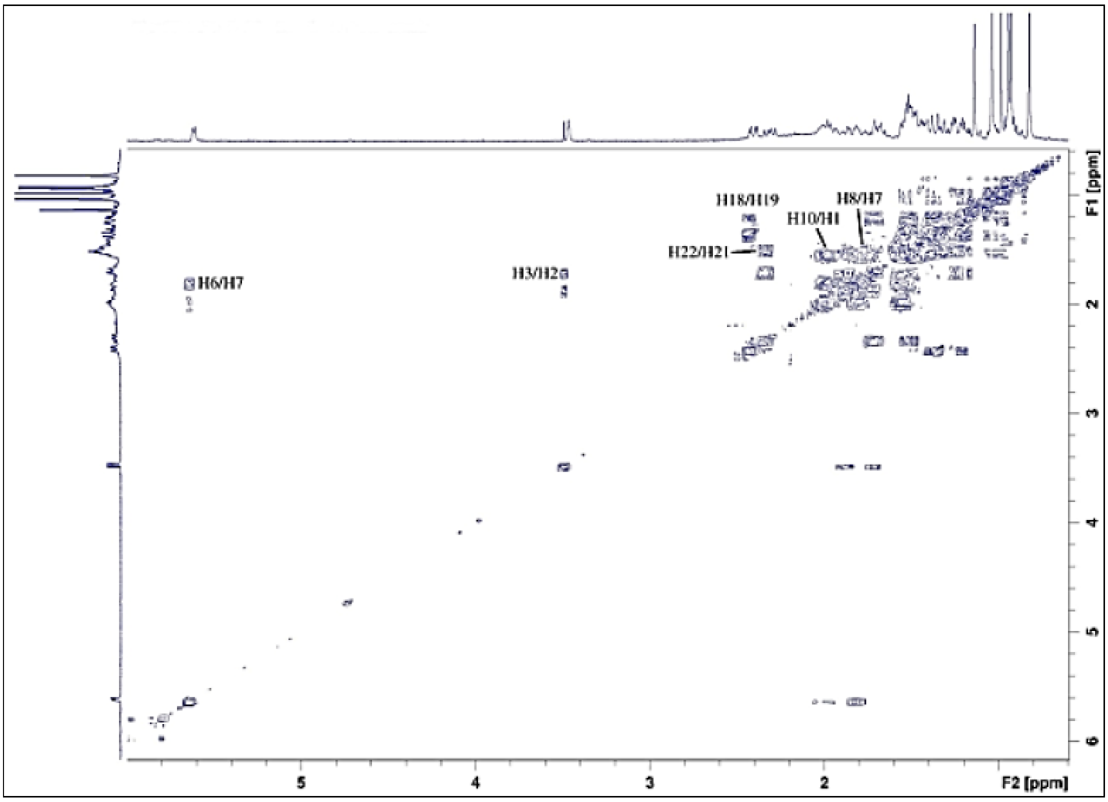

Figure S4 Homonuclear correlation spectroscopy (COSY) spectrum of pinnatane A.

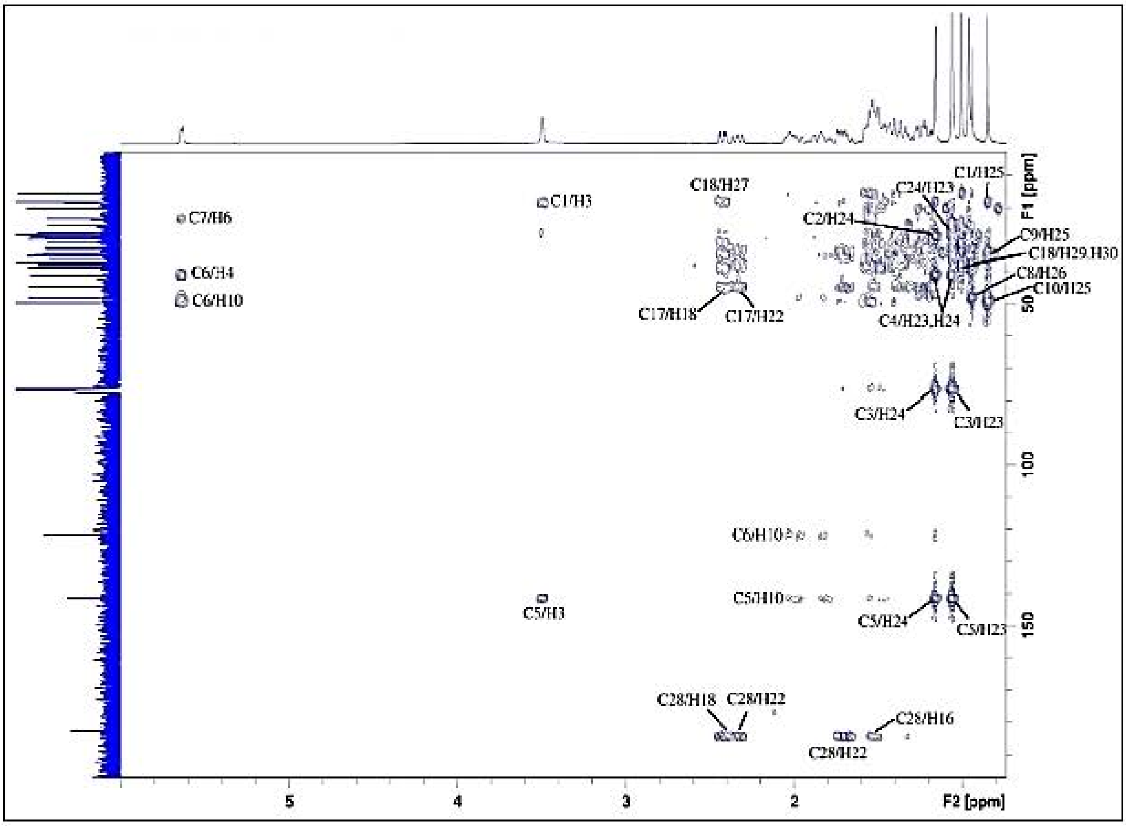

Figure S5: Heteronuclear multiple bond correlation (HMBC) spectrum of pinnatane A.

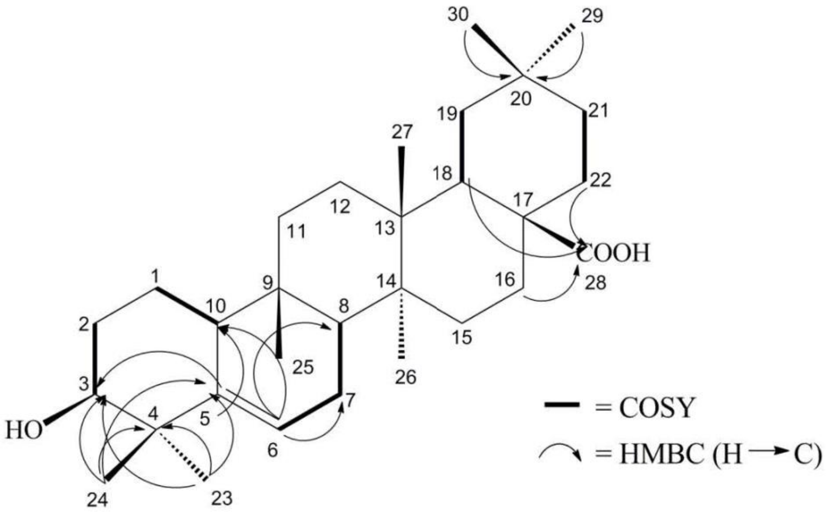

Figure S6: Selected COSY and HMBC correlations of pinnatane A.

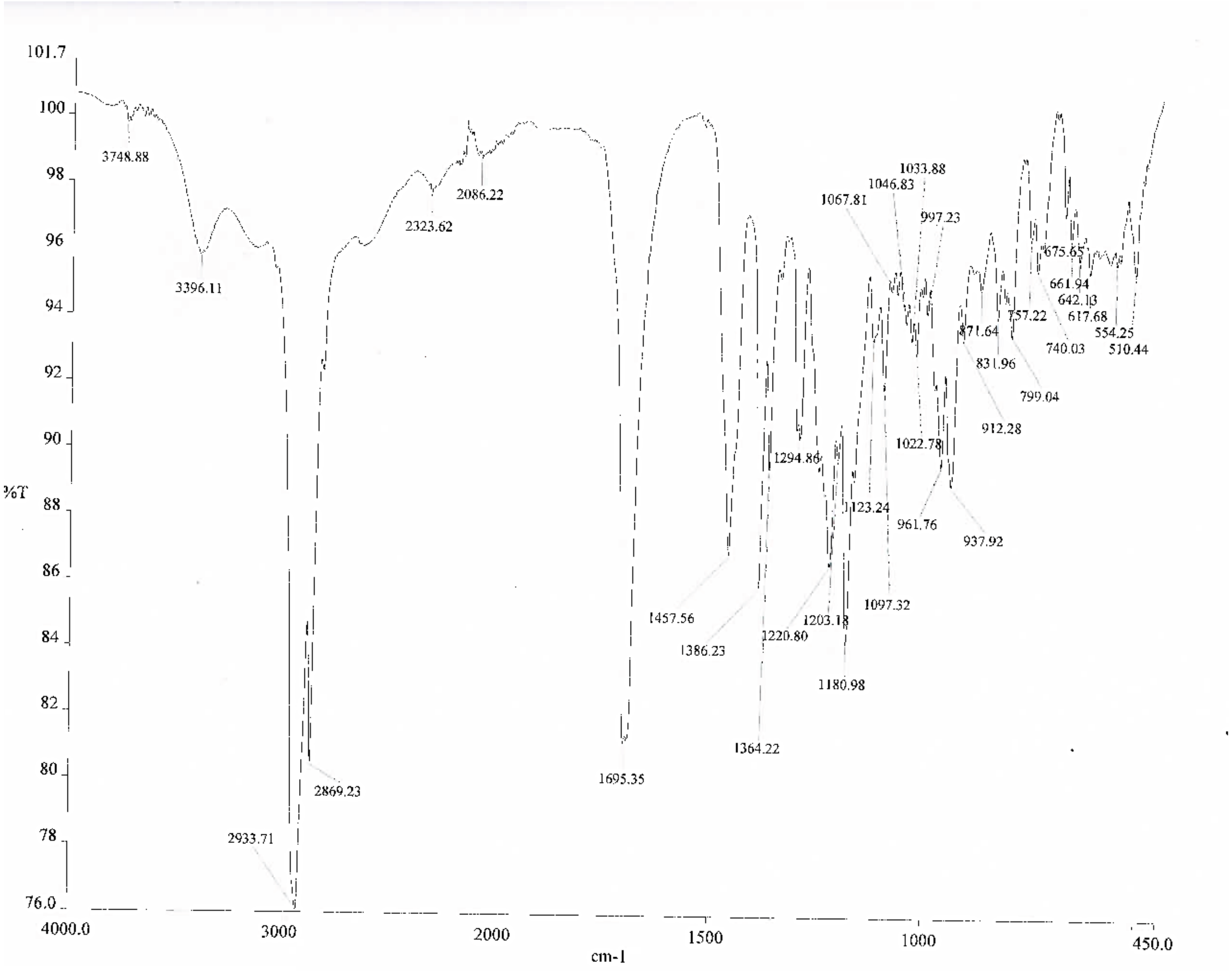

Figure S7: Infrared (IR) spectrum of pinnatane A

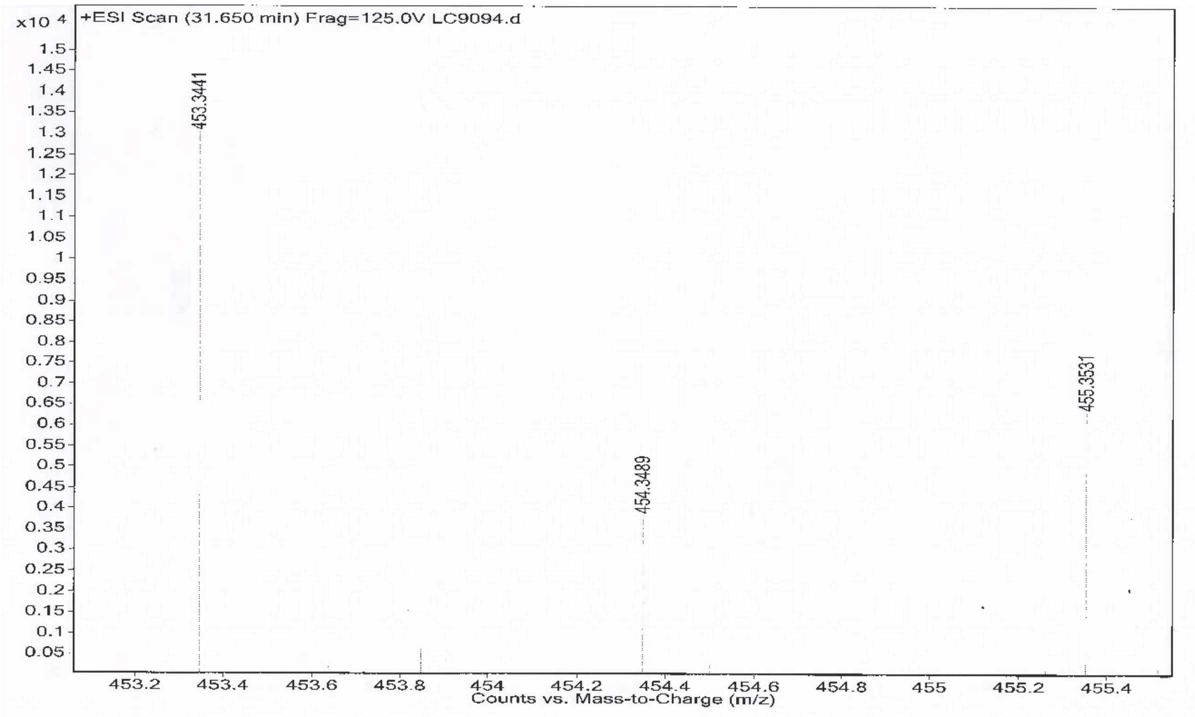

Figure S8: Liquid chromatography–mass spectrometry (LC-MS) spectrum of pinnatane A.

Related references:

1. Elfita, E.; Muharni, M.; Latief, M.; Darwati, D.; Widiyantoro, A.; Supriyatna, S.; Bahti, H.H.; Dachriyanus, D.; Cos, P.; Maes, L., et al. Antiplasmodial and other constituents from four Indonesian *Garcinia* spp. Phytochemistry, 2009. 70(7): pp. 907-12. DOI: 10.1016/j.phytochem.2009.04.024.
2. Mohamad, K.; Yusoff, M.; Awang, K.; Ahmad, K.; Ng, S.W. Pinnatane A from the bark of *Walsura pinnata* Hassk. Acta Crystallogr Sect E Struct Rep Online, 2009. 65(Pt 6): pp. o1317. DOI: 10.1107/s1600536809015955.
3. Yusoff, M. Chemical constituents of *Walsura pinnata* Hassk., in Department of Chemistry. 2012, University of Malaya, Kuala Lumpur, Malaysia (Dissertation). url: http://studentsrepo.um.edu.my/5775/ (accessed on 15 August 2018). pp. 96-99.
4. Mahdzir, M.A.; Shilpi, J.A.; Mahmud, N.; Ramasamy, S.; Awang, K. Chemical constituents from *Walsura pinnata* (Meliaceae). Nat Prod Commun, 2017. 12(9): pp. 1397-1400.
